# Computational Advances in Taste Perception: From Ion Channels and Taste Receptors to Neural Coding

**DOI:** 10.3390/brainsci16010010

**Published:** 2025-12-22

**Authors:** Vladimir A. Lazovsky, Sergey V. Stasenko, Roman K. Khismatullin, Victor B. Kazantsev

**Affiliations:** 1 Moscow Center for Advanced Studies, Kulakova Str. 20, 123592 Moscow, Russia; 2 Scientific and Educational Mathematical Centre ‘Mathematics for Future Technologies’, Lobachevsky State University of Nizhny Novgorod, Gagarin Avenue 23, 603022 Nizhny Novgorod, Russia; 3 Institute of Neurosciences, Lobachevsky State University of Nizhny Novgorod, Gagarin Avenue 23, 603022 Nizhny Novgorod, Russia

**Keywords:** gustatory system, biophysical neuron models, spike-timing-dependent plasticity, multiscale neural coding

## Abstract

We present a multiscale model of taste that is both biophysically faithful and computationally efficient, enabling end-to-end simulation from receptor transduction to network-level coding. The novelty lies in coupling Hodgkin–Huxley taste receptor cells with Goldman–Hodgkin–Katz ion currents and modality-specific receptors (T1R/T2R, ENaC), to an Izhikevich spiking network equipped with realistic glutamatergic synapses and spike-timing-dependent plasticity. Training combines spike synchrony and a genetic approach in order to reach both globally optimized network structure and biomorphic synaptic plasticity. This hybrid design yields distinct, sparse spiking “fingerprints” for taste qualities and mixtures, and provides a practical foundation for neuromorphic gustatory sensors that require real-time, energy-efficient operation.

## 1. Introduction

The gustatory system plays a crucial role in detecting and processing taste stimuli, enabling the perception of five primary taste modalities: sweet, sour, bitter, salty, and umami [1]. Taste perception begins when chemical compounds dissolve in saliva and interact with taste buds, clusters of 50 to 100 specialized sensory cells primarily located on the tongue [1]. These cells are categorized into three main types based on their structure and function. Type I cells provide metabolic and structural support and are implicated in detecting salty stimuli. Type II cells act as receptor cells, responding to sweet, bitter, and umami tastes via G protein-coupled receptors (GPCRs). Type III cells are presynaptic cells that primarily sense sour stimuli and transmit signals to sensory neurons [1].

The molecular mechanisms underlying taste perception are modality-specific. Sweet and umami tastes are detected by heterodimeric receptors from the TAS1R family: TAS1R2/TAS1R3 for sweet and TAS1R1/TAS1R3 for umami [1,2]. Bitter perception is mediated by a diverse family of approximately 30 TAS2R receptors [3]. A salty taste is believed to involve epithelial sodium channels (ENaCs), while a sour taste relies on proton-sensitive channels [1,4]. For sweet, bitter, and umami tastes, receptor activation initiates a signaling cascade involving the G protein gustducin, leading to the production of inositol triphosphate (IP_3_), subsequent intracellular calcium release, and finally, the release of ATP as a neurotransmitter through the CALHM1 ion channel. These signals are then relayed to the central nervous system via cranial nerves [1].

The mathematical modeling of the gustatory system faces significant challenges, including the complex, multisensory nature of flavor perception, which integrates taste with olfaction, texture, and temperature [1]. Current modeling approaches can be broadly divided into two categories: biophysically detailed models and neuromorphic systems. For instance, Tateno et al. (2007) proposed a computational model of taste buds using leaky integrate-and-fire neurons to demonstrate that noise-induced synchronization could serve as a mechanism for taste detection [5]. Soltic et al. (2008) developed an evolving spiking neural network (ESNN) with leaky integrate-and-fire (LIF) neurons that used rank-order population coding to mimic biological taste discrimination, emphasizing incremental learning and synaptic plasticity [6]. Other approaches are less focused on biological mimicry. Lvova et al. 2002 created a portable electronic tongue using chemical sensors combined with statistical methods like Principal Component Analysis (PCA) to achieve high classification accuracy for beverages, but without direct biological correlation [7]. More recently, deep learning has been applied, with Dutta et al. (2023) using Deep and Graph Neural Networks (DNNs and GNNs) to classify molecules as bitter, sweet, or umami directly from their structure, showcasing the potential of AI for tastant discovery [8]. These existing models often sacrifice biophysical realism for computational efficiency or vice versa, highlighting a gap in the field.

Our work sets three concrete objectives to bridge biophysical fidelity and scalable computation in gustatory modeling:

1. To establish a biologically grounded transduction layer by implementing biomorphic taste cells with modality-specific receptor dynamics (T1R/T2R for sweet and bitter; ENaC for salty; H^+^ channels for sour) and Goldman–Hodgkin–Katz ion-current calculations, with the goal of preserving ionic realism required for end-to-end simulation of taste transduction and neural coding.
2. To construct mechanistic synapses by modeling glutamate release with alpha-function kinetics and phosphorylation-dependent AMPA receptor trafficking coupled to spike-timing-dependent plasticity, with the goal of enabling adaptive, temporally precise coding at the synaptic level.
3. To develop a multiscale learning framework that jointly optimizes temporal spike synchrony and combinatorial population patterns, with the goal of producing distinct, sparse, and reproducible spiking fingerprints for pure and mixed tastants and supporting efficient large-scale simulation via Hodgkin–Huxley and Izhikevich single-cell dynamics.

Together, these aims yield a hybrid framework that is both biologically plausible and computationally efficient. This provides a practical foundation for developing real-time, energy-efficient neuromorphic electronic tongues and enables future work on cross-modal integration to model the full complexity of flavor perception [1].

## 2. Materials and Methods

### 2.1. Computational Environment

All simulations and analyses were performed on a dedicated workstation to ensure reproducibility and performance transparency. The workstation was equipped with an 11th Gen Intel Core i5-11400 processor running at 2.60 GHz (base 2.59 GHz), 32 GB of installed RAM, and a 64-bit operating system on an ×64-based architecture.

To avoid confounds due to hardware variability, all core experiments (training, inference, and parameter sweeps) were executed on this single system; stochastic components (e.g., randomized initial conditions) were controlled via fixed seeds. The operating system was 64-bit; all compiled and interpreted dependencies were built for ×64 to match the processor architecture.

### 2.2. Modeling of Individual Taste Cells

The taste cell was modeled as a computational unit integrating four types of taste detectors, each corresponding to a distinct taste modality:

- **Sweet**—detection via heterodimeric class C GPCRs *T1R2/T1R3* in Type II taste receptor cells [1,2,3]. Model interpretation: modeled as a metabotropic receptor pathway driving downstream conductances and transmitter release (reduced GPCR cascade) in line with the simplified single-cell block [1,2].
- **Bitter**—detection via *TAS2R* (*T2R*) GPCR family in Type II cells [2,3,9]. Model interpretation: implemented as a metabotropic receptor pathway analogous to sweet, with receptor-specific activation and reduced second-messenger kinetics [2,9].
- **Salty**—Salt taste engages at least two partially dissociable transduction branches that differ by concentration range, pharmacology, and behavioral valence. Appetitive, amiloride-sensitive responses at low–moderate NaCl are mediated by epithelial sodium channels (*ENaC*) on taste-bud cells associated with the Type I lineage, where Na^+^ influx along the electrochemical gradient depolarizes the cell and promotes ATP release to gustatory afferents [1,10]. Aversive, amiloride-insensitive responses to high salt reflect anion-dependent mechanisms and additional ion-channel pathways (including inhibition of K^+^ conductances and TRP-like contributions), with cellular participation extending beyond the ENaC pathway and involving presynaptic Type III circuitry; these routes can vary across anions and produce distinct molecular dynamics and behavioral outcomes [1]. Model interpretation: in this work, the salty pathway is instantiated specifically for sodium chloride as an ENaC-like, non-voltage-gated Na^+^ conductance (GHK-driven), capturing appetitive and mid-range NaCl responses; amiloride pharmacology is not explicitly simulated, anion-specific high-salt (amiloride-insensitive) mechanisms are omitted, and receptor-level contributions reported for certain organic or chloride-containing tastants are outside the present scope [1,10].
- **Sour**—detection primarily in Type III presynaptic cells via proton-sensitive mechanisms that depolarize the cell (including H^+^ conductance and inhibition of K^+^ channels), followed by classical synaptic transmission (serotonin, GABA) to afferents [11,12]. Model interpretation: implemented as a non-voltage-gated H^+^ (proton) depolarizing pathway (GHK-driven current) with reduced channel specificity, preserving the net H^+^-dependent depolarization and synaptic output [11,12].
- **Umami** as an independent taste modality is absent in our model. From a computational neuroscience perspective, umami shares the same metabotropic transduction mechanism as sweet and bitter tastes [13]. Specifically, umami receptors (T1R1/T1R3 heterodimers) utilize G-protein coupled signaling cascades and intracellular IP_3_/Ca^2+^ pathways, described by the same Hill-type kinetics (Equations (3) and (4)) as sweet (T1R2/T1R3) and bitter (T2R) receptors. At the level of our model’s equations, these three modalities differ only in the ligand concentration [L] and binding affinity $K_{D}$, but the functional form of the transduction current $I_{meta}$ is identical. Moreover, as noted in recent comprehensive reviews [14,15], umami compounds constitute a significantly smaller chemical space compared to sweet or bitter tastants, with most databases containing fewer than 800 umami molecules [16], predominantly short peptides (glutamate, nucleotides). This narrow chemical domain would not provide sufficient diversity to test the network’s plasticity mechanisms, which were optimized for discriminating broader taste categories. Therefore, we focused our validation on the three major metabotropic classes (sweet, bitter) plus the ionotropic modalities (salty, sour), reserving umami integration for future work when receptor-specific affinity data become more widely available.

#### 2.2.1. Ionic Current Calculations

1. **Electrodiffusion currents (Salty/Sour).** The current through Na^+^ and H^+^ channels was computed using the **Goldman–Hodgkin–Katz (GHK)** equation for the reversal potential ($E_{x}$) of each ion [17]:
  (1) $$E_{x}=\frac{RT}{zF}\ln\left(\frac{{\left[X\right]}_{\mathrm{out}}}{{\left[X\right]}_{\mathrm{in}}}\right)$$
  where ${\left[X\right]}_{\mathrm{out}}$ and ${\left[X\right]}_{\mathrm{in}}$ are extracellular and intracellular ion concentrations, respectively, *R* is the gas constant, *T* is temperature, *z* is ion valence, and *F* is Faraday’s constant.
  The resultant current ($I_{x}$) was calculated as follows:
  (2) $$I_{x}=g_{x}\left(V_{m}-E_{x}\right),$$
  where $g_{x}$ is the channel conductance and $V_{m}$ is the membrane potential.
2. **Metabotropic currents (Sweet/Bitter).** The activation kinetics of metabotropic receptors (T1R2+T1R3, T2R) were modeled using **Hill equations**, where the fraction of open channels (*O*) depended on tastant concentration ([L]) and the half-maximal activation constant ($K_{a}$), derived from experimentally reported minimal detectable concentrations [18]:
  (3) $$O\left(t\right)=\frac{{\left[L\right]}^{n}}{{\left[L\right]}^{n}+K_{a}^{n}}.$$
  The resulting current ($I_{\mathrm{meta}}$) was computed assuming the reversal potential matched that of voltage-gated Na^+^ channels ($E_{\mathrm{Na}}$):
  (4) $$I_{\mathrm{meta}}=g_{\mathrm{meta}}O\left(t\right)\left(V_{m}-E_{\mathrm{Na}}\right).$$

#### 2.2.2. Integration into Electrophysiological Dynamics

The total membrane current ($I_{\mathrm{tot}}$) generated by taste receptors was the sum of all ionic and metabotropic contributions:

(5) $$I_{\mathrm{tot}}=I_{\mathrm{Na}}+I_{H}+I_{\mathrm{sweet}}+I_{\mathrm{bitter}}.$$

The cellular response was simulated using a hybrid **Hodgkin–Huxley (HH)** and **Izhikevich model**, with membrane potential dynamics governed by the following:

(6) $$C_{m}\frac{dV_{m}}{dt}=I_{\mathrm{tot}}+I_{\mathrm{pd}},$$

where $C_{m}$ is membrane capacitance and $I_{\mathrm{pd}}$ represents the current through potential-dependent channels (as in the classical model). Spiking behavior was further refined using Izhikevich’s reduced model for action potential generation.

#### 2.2.3. Numerical Integration

The differential equations were solved using standard numerical methods:

- **Fourth-order Runge–Kutta (RK4)** with 1 ms step for HH dynamics,
- **Forward Euler** for Izhikevich spiking kinetics.

### 2.3. Postsynaptic Neuron Model

#### 2.3.1. Spike Train Generation

The membrane potential response V(t) of a taste cell was converted into a discrete spike train. Time was partitioned into 5 ms bins, and a binary vector $S\in F_{2}$ was constructed such that

(7) $$S_{k}=\left\{\begin{array}{cc} 1 & \mathrm{if}\;\exists t\in \left[k\Delta t,\left(k+1\right)\Delta t\right)\;\mathrm{where}\;V\left(t\right)\geq V_{\mathrm{threshold}},\\ 0 & \mathrm{otherwise}, \end{array}\right.$$

where Δt=5 ms and $V_{\mathrm{threshold}}$ is the spike detection threshold.

#### 2.3.2. Synaptic Conductance Dynamics

1. **Alpha Function for Glutamate Release.** The time-dependent profile of glutamate release was modeled using a rectified alpha function [19]:
  (8) $$\alpha \left(t\right)=\left\{\begin{array}{cc} \frac{t}{\tau }\exp\left(1-\frac{t}{\tau }\right) & \mathrm{if}\;t\geq 0,\\ 0 & \mathrm{otherwise}, \end{array}\right.$$
  where τ is the time constant of glutamate emission.
2. **Glutamate Concentration in the Synaptic Cleft.** The total glutamate concentration G(t) at time *t* was computed as follows:
  (9) $$G\left(t\right)=A\sum_{j}\alpha \left(t-t_{j}\right),$$
  where *A* is the amplitude of glutamate emission per spike (Table 1), and $\left\{t_{j}\right\}$ are the presynaptic spike times.
3. **Synaptic Conductance $g_{\mathrm{syn}}$.** The total synaptic conductance incorporated phosphorylation-dependent AMPA receptor modulation [20]:
  (10) $$g_{\mathrm{syn}}\left(t\right)=\sum_{i=1}^{N}\left(\frac{g_{\mathrm{AMPA}}\;\cdot \;N_{\mathrm{total},i}\;\cdot \;{\mathrm{Phos}}_{i}\;\cdot \;G_{i}\left(t\right)}{G_{i}\left(t\right)+K_{\mathrm{Phos}}}+\frac{g_{\mathrm{AMPA}}\;\cdot \;N_{\mathrm{total},i}\;\cdot \;\left(1-{\mathrm{Phos}}_{i}\right)\;\cdot \;G_{i}\left(t\right)}{G_{i}\left(t\right)+K_{\mathrm{unPhos}}}\right),$$
  where
  - $g_{\mathrm{AMPA}}$: Maximal conductance of an AMPA receptor (Table 1),
  - $N_{\mathrm{total},i}$: Total number of AMPA receptors at synapse *i*,
  - ${\mathrm{Phos}}_{i}$: Phosphorylation rate (∈[0,1]), i.e the portion of phosphorylated receptors at synapse *i* (Table 1),
  - $K_{\mathrm{Phos}}$, $K_{\mathrm{unPhos}}$: Michaelis constants for phosphorylated/unphosphorylated states of the receptor (Table 1),
  - $G_{i}\left(t\right)$: Glutamate concentration at synapse *i*.

#### 2.3.3. Synaptic Current Integration

The synaptic current $I_{\mathrm{syn}}$ was computed as follows:

(11) $$I_{\mathrm{syn}}\left(t\right)=g_{\mathrm{syn}}\left(t\right)\left(V\left(t\right)-E_{\mathrm{AMPA}}\right),$$

with $E_{\mathrm{AMPA}}$ being the AMPA receptor reversal potential. This was added to the membrane current in the Hodgkin–Huxley/Izhikevich framework:

(12) $$C_{m}\frac{dV}{dt}=-\left(I_{\mathrm{ion}}+I_{\mathrm{syn}}\right)+I_{\mathrm{ext}}.$$

### 2.4. Global Network Architecture

#### 2.4.1. Network Structure

Using the described spike encoding and synaptic transmission mechanisms, we constructed a feedforward spiking neural network with the following layers:

- **Input Layer**: Composed of taste cells with varying arrangements of taste detectors (sweet, bitter, salty, sour). Each cell’s output was encoded as a binary spike train $S_{k}\in F_{2}$ using the 5 ms binning procedure described in Section 2.2.
- **Hidden Layers**: Consisting of postsynaptic neurons driven by glutamate-mediated synapses. The synaptic dynamics followed the phosphorylation-dependent conductance model [21]:
  (13) $$g_{\mathrm{syn},i}\left(t\right)=\frac{g_{\mathrm{AMPA}}N_{\mathrm{total},i}G_{i}\left(t\right)}{G_{i}\left(t\right)+K_{\mathrm{eff}}}$$
  where $K_{\mathrm{eff}}=K_{\mathrm{Phos}}$ (if phosphorylated) or $K_{\mathrm{unPhos}}$ (otherwise), implementing activity-dependent plasticity.

**Figure A1:**
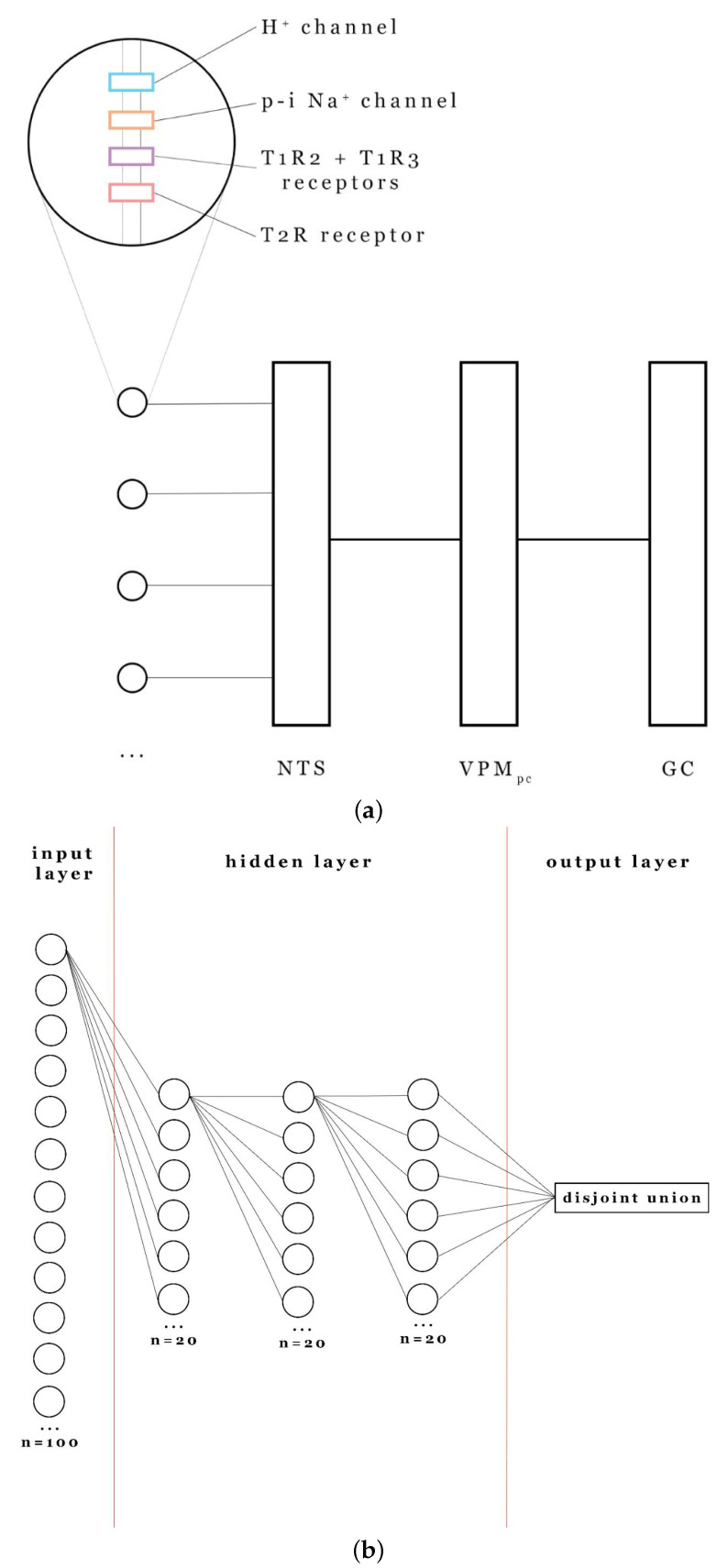
(**a**) Scheme of the taste analyzer. (**b**) Biological analyzer vs. model structure.

The network has 3 hidden layers with 20 neurons in each one. Every neuron is initially connected with all the neurons of the next layer. All the connections are one-sided. The disjoint union of the spike series of all the neurons of the last hidden layer is considered as the network’s output and is used in computing the loss function (see Section 2.5). The network architecture is depicted in Figure A1.

#### 2.4.2. The Multiscale Integration Approach

The integration mechanism works as follows: biophysical Hodgkin–Huxley dynamics at the receptor level generate total ionic currents $I_{tot}$ (Equation (5)), which drive the membrane potential $V_{m}$ (Equation (6)) until spike threshold is reached. These spikes are then converted into discrete binary vectors (Equation (7)) and transmitted to the downstream Izhikevich network via glutamatergic synapses with phosphorylation-dependent conductances (Equations (10)–(12)).

This hybrid design preserves the non-linear concentration–response properties of peripheral transduction—such as receptor saturation and adaptation—while enabling scalable simulation of network-level plasticity. Critically, the sparse and structured “neural fingerprints” observed after training (Figure A6 and Figure A8) would not emerge without the temporal precision inherited from the HH layer: the millisecond-scale dynamics of GHK currents (Equations (1) and (2)) and metabotropic kinetics (Equations (3) and (4)) produce graded receptor potentials that translate into precise spike timing in the network. This contrasts with fully abstract models where input stimuli are pre-encoded as rate codes, bypassing the rich dynamics of ionic transduction [22,23]. Thus, the multiscale integration is not merely a modeling choice, but a functional requirement for capturing how molecular-level deficits (e.g., ENaC mutations) propagate to impaired taste discrimination at the network level.

#### 2.4.3. Synaptic Weight Representation and Plasticity

To ensure biological plausibility, synaptic weights in the network were governed by two physiologically grounded parameters that reflect known molecular mechanisms of long-term potentiation (LTP):

1. **Total AMPA receptors** ($N_{\mathrm{total},i}$): This parameter determines the baseline synaptic strength by representing the number of AMPA-type glutamate receptors available at the postsynaptic density. Synaptic efficacy is fundamentally limited by receptor availability [24].
2. **Phosphorylation state** (${\mathrm{Phos}}_{i}$): This variable models the activity-dependent modulation of receptor conductance via phosphorylation of AMPA receptor subunits (e.g., GluA1 at Ser845), a well-established cellular mechanism underlying spike-timing-dependent plasticity (STDP) and LTP [21]. The phosphorylation dynamics were updated according to a spike-based Hebbian learning rule:
  (14) $$\Delta {\mathrm{Phos}}_{i}=\eta \;\cdot \;\sum_{j}S_{j}\;\cdot \;\left(1-{\mathrm{Phos}}_{i}\right)/N,$$
  where
  - η: Learning rate (fixed at 0.05), representing the timescale of biochemical cascades
  - $S_{j}$: Synchronization index for spike train pair *j*, quantifying the temporal correlation between pre- and postsynaptic activity [25]:
    $$S_{j}=0.5-\frac{d_{j}+\Delta t}{D}$$
    where
    − $d_{j}$: Minimal interspike interval between pre- and postsynaptic neurons in trial *j*
    − Δt: Temporal resolution bin size (5 ms)
    − *D*: Maximum observed interspike interval across all trials.
  - *N*: The number of spike train pairs.

This formulation captures the saturation property of LTP ($1-{\mathrm{Phos}}_{i}$ term), reflecting the finite capacity of receptor phosphorylation [26,27], and the dependence on spike timing precision ($S_{j}$ term), consistent with experimental observations of STDP time windows [28] and AMPA receptor phosphorylation-dependent plasticity [24].

#### 2.4.4. Output Encoding

The network produced a k×m-dimensional output vector over response period T=300 ms:

$$Y\in F_{2}^{k\times m},\;k=\left\lfloor \frac{T}{\Delta t}\right\rfloor$$

where

- k=60 bins (Δt=5 ms),
- *m*: Number of output neurons,
- Each entry $Y_{i,j}$ indicated a spike in neuron *j* during bin *i*.

#### 2.4.5. Information Flow

1. Taste inputs → Spike trains via receptor dynamics (Modeling of individual taste cells subsection),
2. Spike trains → Synaptic currents (Postsynaptic neuron model subsection),
3. Currents → Output spikes through iterative membrane potential updates.

### 2.5. Learning

#### Genetic Optimization

The network’s synaptic weights were optimized through an evolutionary approach:

- **AMPA** Receptor Counts $N_{\mathrm{total},i}$: Optimized using a genetic algorithm with. This process functionally mimics homeostatic synaptic scaling, setting the maximal synaptic capacity over long timescales [29].
  − Population size: 100 individuals
  − Selection: Rank-based, keeping top 50% performers
  − Mutation: Gaussian noise ($\sigma =0.1N_{\mathrm{total},i}$)
  − Crossover: Uniform mixing of parent parameters.
- **Phosphorylation Rates (${\mathrm{Phos}}_{i}$)**: Adapted online according to synaptic activity [24]:
  $${\mathrm{Phos}}_{i}^{\left(n+1\right)}={\mathrm{Phos}}_{i}^{\left(n\right)}+\Delta {\mathrm{Phos}}_{i}^{\left(\mathrm{top}\;50\%\right)}$$
  where $\Delta {\mathrm{Phos}}_{i}^{\left(\mathrm{top}\;50\%\right)}$ is the mean $\Delta {\mathrm{Phos}}_{i}$ in the best-performing individuals.

### 2.6. Parameter Values

All model constants were derived from experimental data or calibrated through simulation:

- Temperature: T=310 K (physiological)
- Bin width: Δt=5 ms (temporal resolution for PSTH and spike-train metrics, cf. [30])
- Simulation time: $T_{\mathrm{sim}}=300\;\mathrm{ms}$ per trial (typical duration for STDP protocols, cf. [25]).

**Table 1:** Key Model Parameters with Supporting Literature.

Parameter	Value	Description and Source
τ	2.5 ms	Glutamate emission time constant (alpha-function synapse kinetics) [4,31]
*A*	1.2 mM	Glutamate release amplitude (peak cleft concentration) [4,31]
$g_{\mathrm{AMPA}}$	10 pS	Single AMPA receptor conductance (unitary channel) [32,33]
$K_{\mathrm{Phos}}$	0.3 mM	Phosphorylated AMPA Michaelis constant (fitted from phosphorylation kinetics) [20,34]
$K_{\mathrm{unPhos}}$	1.5 mM	Unphosphorylated AMPA Michaelis constant (fitted from phosphorylation kinetics) [20,34]
$E_{\mathrm{AMPAR}}$	0 mV	AMPA reversal potential (cation-nonselective channel) [35,36]
η	0.05	Phosphorylation learning rate (STDP scaling parameter) [37,38,39]
$V_{\mathrm{threshold}}$	−50 mV	Spike detection threshold (HH model threshold) [40,41]

### 2.7. Tasks

The learning process was divided into optimizing the model’s opportunity of both dividing tastants by their pleasantness and discriminating different tastes.

#### 2.7.1. Task 1: Encoding Taste Pleasantness

The loss function quantified the preservation of hedonic distance between stimuli:

(15) $$L_{1}=\sum_{i=1}^{n}-\frac{|D_{\mathrm{in}}\left(A_{i},B_{i}\right)-D_{\mathrm{res}}\left(A_{i},B_{i}\right)|}{n}$$

where

- $D_{\mathrm{taste}}\left(A,B\right)$: Distance in taste pleasantness space for stimuli $A=(A_{\mathrm{Na}},A_{H},A_{S},A_{Q})$ and $B=(B_{\mathrm{Na}},B_{H},B_{S},B_{Q})$:
  (16) $$D_{\mathrm{taste}}\left(A,B\right)=\left|\prod_{i\in \{\mathrm{Na},H,S,Q\}}\exp\left(-\frac{{\left(A_{i}-\theta_{i}\right)}^{2}}{w_{i}^{2}}\right)-\prod_{i\in \{\mathrm{Na},H,S,Q\}}\exp\left(-\frac{{\left(B_{i}-\theta_{i}\right)}^{2}}{w_{i}^{2}}\right)\right|$$
  where
  − $\theta_{\mathrm{Na}}=100.0$, $w_{\mathrm{Na}}$ = 50.0 (optimal NaCl concentration and width)
  − $\theta_{H}=0.1$, $w_{H}=2.0$ (optimal acidity and width)
  − $\theta_{S}=15000.0$, $w_{S}$ = 10,000.0 (optimal sweetness and width)
  − $\theta_{Q}=1.0$, $w_{Q}=5.0$ (optimal bitterness and width).
- $D_{\mathrm{res}}$: Information distance between output spike trains:
  (17) $$D_{\mathrm{res}}\left(A,B\right)=H\left(X,Y\right)-MI\left(X;Y\right)$$
  with *H* as joint entropy and MI as mutual information.

#### 2.7.2. Task 2: Taste Discrimination

For pure vs. mixed taste differentiation, the input metric was as follows:

(18) $$D_{\mathrm{in}}\left(A,B\right)=\left\{\begin{array}{cc} {∥A∥}_{1}+{∥B∥}_{1} & \mathrm{if}\;\mathrm{both}\;\mathrm{pure}\;\mathrm{tastes}\\ \frac{1}{2}{∥A-B∥}_{2} & \mathrm{otherwise} \end{array}\right.$$

with output distance $D_{\mathrm{res}}$ computed as in Task 1. The loss:

(19) $$L_{2}=\sum_{i=1}^{n}-\frac{|D_{\mathrm{in}}\left(A_{i},B_{i}\right)-D_{\mathrm{res}}\left(A_{i},B_{i}\right)|}{n}.$$

## 3. Results

Firstly, we need to note that training was performed only for the Izhikevich version of the model due to its computational efficiency compared to the full Hodgkin–Huxley dynamics. Therefore, all subsequent results and discussions pertain to the Izhikevich-based network.

### 3.1. Experimental Procedure and Network Evaluation

To evaluate the network’s ability to encode and discriminate taste stimuli, we conducted a series of computational experiments. Each experiment involved presenting the model with a specific taste stimulus, represented as a vector of four concentrations corresponding to the primary taste modalities: salty ($\left[Na^{+}\right]$), sour ($\left[H^{+}\right]$), sweet, and bitter.

As described in Section 2 these input concentrations modulate the ionic currents in the input layer neurons, causing changes in their membrane potentials. When a neuron’s potential crosses the firing threshold ($V_{\mathrm{threshold}}$), it generates a spike. The collective spiking activity of all neurons across all layers was simulated for a period of 300 ms for each input stimulus. The resulting output of the model for a given stimulus is a set of spike raster plots, which show the precise timing of spikes for each neuron in the network over the simulation period. The training process, driven by a genetic algorithm, aimed to optimize the network’s synaptic weights to produce distinct and informative output spike patterns for different tastes, based on the specific loss functions defined for each task.

### 3.2. Untrained Network Responses

**Figure A2:**
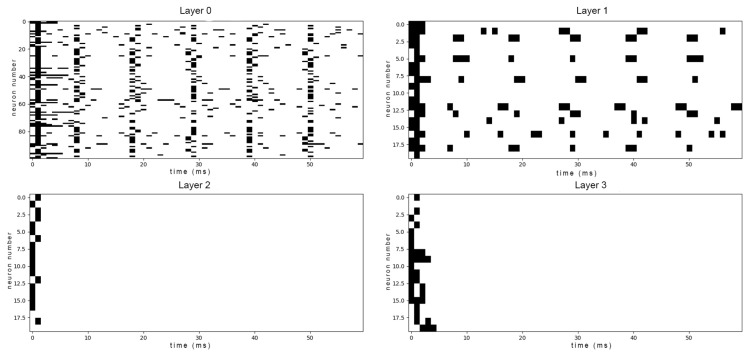
Raw output spike patterns of all the untrained network’s layers to a mid-concentrated salty input. Each row shows the spike raster of a taste cell population over 300 ms.

**Figure A3:**
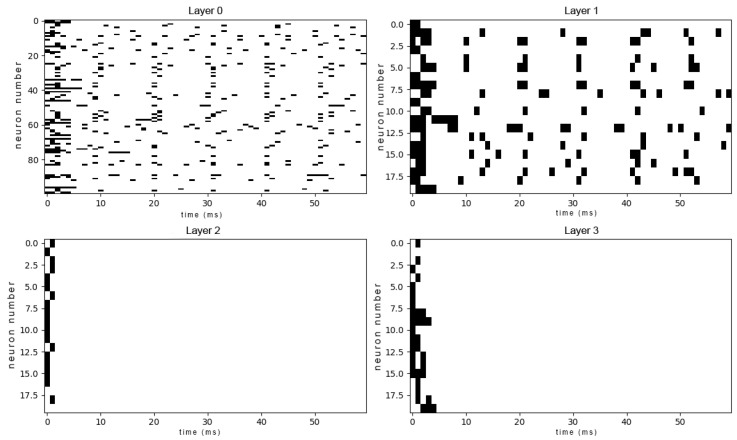
Raw output spike patterns of all the untrained network’s layers to a high-concentrated mixed sweet–sour input. Each row shows the spike raster of a taste cell population over 300 ms.

Before training, we analyzed the network’s baseline activity in response to taste stimuli. Figure A2 and Figure A3 show the spike raster plots for all layers of the untrained network when presented with a mid-concentration salty input and a high-concentration mixed sweet–sour input, respectively. The activity across all layers appears dense, noisy, and lacks clear, discernible patterns. Moreover we cannot see any significant difference between the answers to single-taste and mixed tastants. This unstructured response serves as a control, demonstrating that without optimization, the network is incapable of forming a specific representation of the taste stimulus.

### 3.3. Learning Dynamics

The network was trained on two distinct tasks: encoding the perceived pleasantness (hedonic value) of tastes and discriminating between pure and mixed taste stimuli. The learning progress was tracked by monitoring the fitness function, which corresponds to the negative of the loss function, maximized by the genetic algorithm.

**Figure A4:**
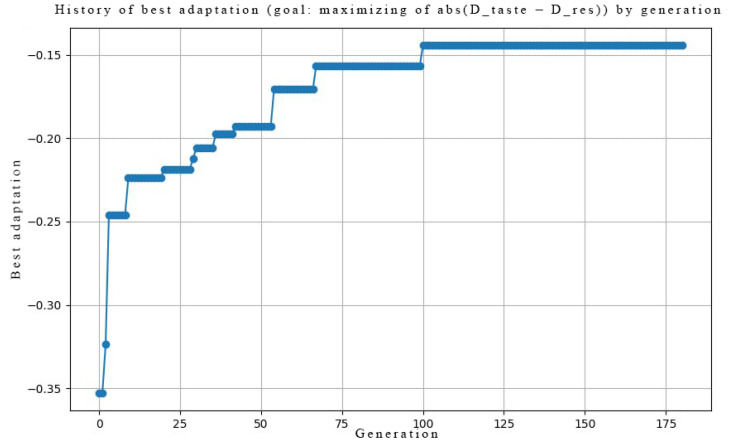
Evolution of fitness (maximizing $|D_{\mathrm{taste}}-D_{\mathrm{res}}|$) across generations. Normalized to [−1,0] range.

**Figure A5:**
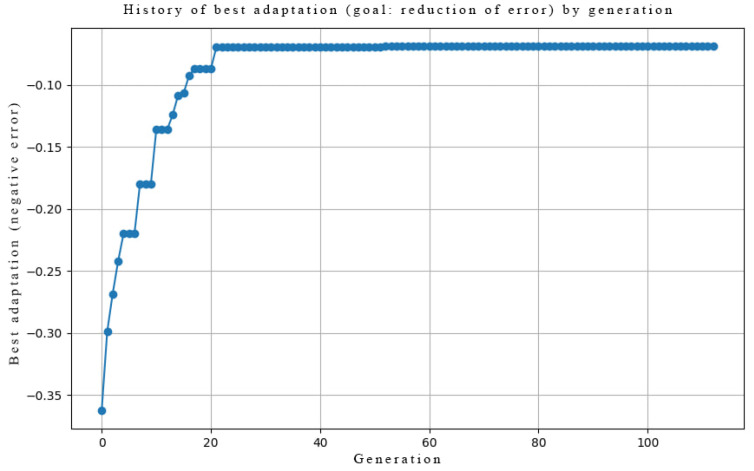
Evolution of fitness (reduction of error) across generations. Normalized to [−1,0] range.

Figure A4 and Figure A5 illustrate the evolution of the best fitness score across generations for Task 1 and Task 2, respectively. In both cases, the fitness value steadily increases and converges to 0.14 and 0.04, respectively, indicating that the genetic algorithm successfully optimized the network’s synaptic parameters (AMPA receptor counts and phosphorylation rates) to minimize the error between the target taste representation and the network’s output spike patterns. This confirms that the model is capable of learning the desired input–output mappings.

### 3.4. Trained Network Responses and Taste Recognition

**Figure A6:**
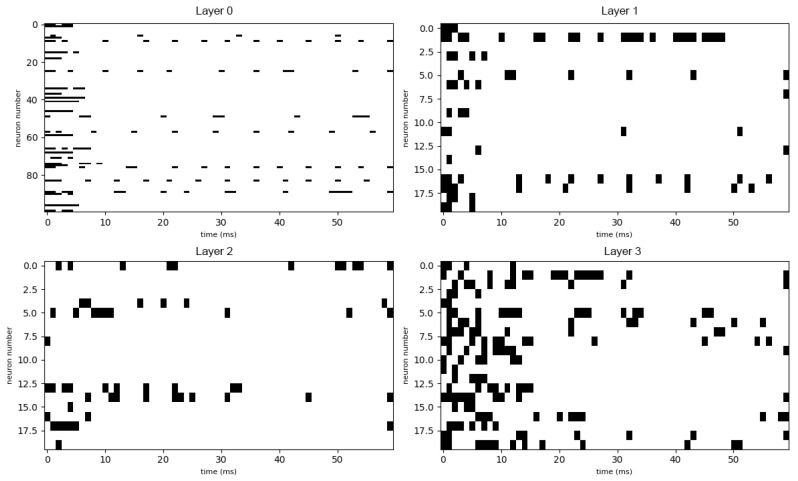
Raw output spike patterns of all the trained network’s layers to a mid-concentrated salty input. Each row shows the spike raster of a taste cell population over 300 ms.

After the training process was completed, the network was re-evaluated using the same input stimuli as in the pre-training phase. The resulting spike patterns for the trained network are shown in Figure A6 (mid-concentration salty) and Figure A8 (high-concentration mixed sweet–sour).

**Figure A7:**
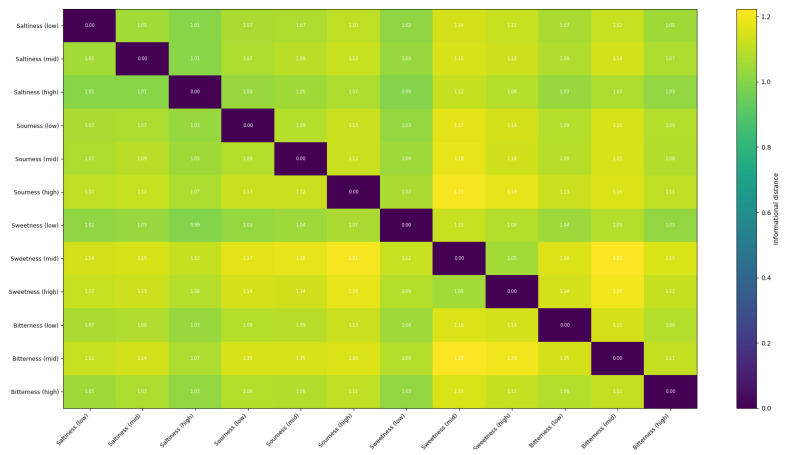
Informational distance between outputs to different tastes of the trained network.

In sharp contrast to the chaotic activity of the untrained network, the responses of the trained network are significantly more sparse, structured, and reproducible. Specific groups of neurons fire in coordinated, temporally precise patterns that are unique to the input stimulus. This transformation from noisy to structured activity demonstrates that the network has learned to form distinct and efficient neural codes for different tastes. Comparing this data to the outputs of the untrained network, we can notice the growth of selectivity especially in the single-taste experiment, so we can conclude that the model has at least been combinatorially tuned on discriminating different tastes. The emergence of these specific output patterns is the basis for taste recognition, as they represent a unique “neural fingerprint” for each stimulus, which can be reliably decoded by downstream neural structures to identify the taste. Also we can see in Figure A7 how the network is discriminating different tastes.

It is instructive to compare the spike raster plots generated by our trained network with electrophysiological recordings from biological gustatory neurons. The sparse, phasic burst patterns observed in our model (e.g., Figure A6) closely resemble the “latency coding” regime reported in the rat geniculate ganglion. Specifically, experimental studies have shown that peripheral taste neurons encode stimulus identity not only through firing rate but through stimulus-specific response latencies and burst durations [42,43]. Similarly, recordings from the nucleus of the solitary tract (NTS) in awake rats reveal that taste information is conveyed via precise temporal sequences of spikes locked to the lick cycle [44,45]. Our model reproduces this key biological feature: before training, responses are dense and unstructured (Figure A2), whereas after optimization, the network converges to a regime of temporally precise, reproducible spike trains similar to those observed in vivo.

**Figure A8:**
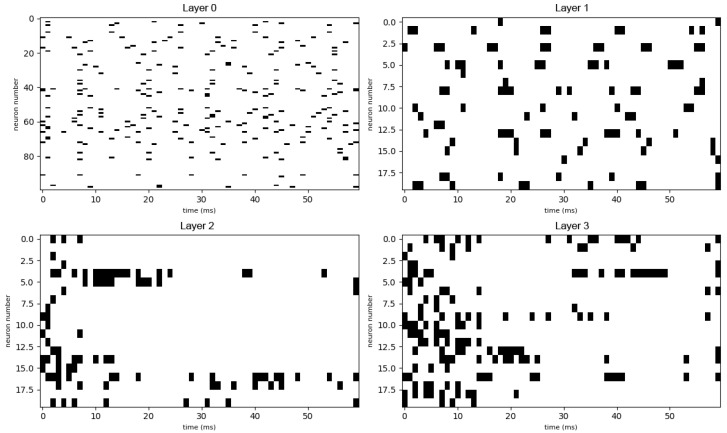
Raw output spike patterns of all the trained network’s layers to a high-concentrated mixed sweet–sour input. Each row shows the spike raster of a taste cell population over 300 ms.

The sparse and temporally structured spike patterns generated by our model (Figure A6 and Figure A8) are consistent with experimental evidence supporting temporal coding in the gustatory system. Electrophysiological recordings in the rat geniculate ganglion have demonstrated that, in addition to spike count, the precise timing and latency of spikes are critical for encoding taste quality [42,43]. Specifically, Breza et al. (2010) [42] showed that distinct response latency profiles allow for the discrimination of tastants even by broadly tuned neurons. Similarly, studies in the nucleus of the solitary tract (NTS) reveal that taste-responsive neurons convey significant information through the temporal structure of spike trains, often distinguishing stimuli that elicit similar overall firing rates [44,45]. The “neural fingerprints” observed in our trained network—characterized by stimulus-specific phasic bursts and reproducible timing—recapitulate these biological coding strategies. This suggests that the optimization process drives the network toward a biologically plausible regime where information is multiplexed in both the spatial (neuron identity) and temporal (spike timing) domains.

### 3.5. Quantitative Analysis of Neural Coding

To quantify the information capacity of the observed spike patterns, we calculated the Shannon entropy of the spike timing distribution [44]. For the optimized network responding to tastants, the spike pattern entropy was calculated using 3 ms time bins over the 60 ms stimulation period. The resulting entropy was H=850 bits (compared to a theoretical maximum of 1200 bits for a uniform distribution), indicating a highly structured temporal code rather than random firing. This high entropy value confirms that the sparse “fingerprints” carry significant temporal information, consistent with the mutual information analyses of taste neurons in the nucleus of the solitary tract [45].

## 4. Discussion

The hybrid model of taste perception presented in this work successfully integrates biological fidelity at the level of ion channels and synaptic transmission with the computational efficiency required for medium-scale network simulations. Our model demonstrates how the optimization of synaptic parameters via a genetic algorithm enables the network to form sparse and structured spike patterns that serve as unique “neural fingerprints” for different taste stimuli. This serves as a proof of principle that biomorphic computing can efficiently encode complex sensory information.

### 4.1. Limitations of the Model

Despite the promising results, our model has several limitations that are important to acknowledge.

1. **Biophysical Simplification:** Although we utilized GHK and Michaelis–Menten equations to describe currents, the model omits many intricacies of intracellular signal transduction. For instance, for bitter, sweet, and umami tastes, the cascades of secondary messengers (IP_3_, DAG), the dynamics of Ca^2+^ release from intracellular stores, and the subtle modulation of voltage-gated channels are not fully modeled. This may limit the accuracy of predicting responses to complex or low-concentration stimuli.
2. **Network Architecture:** The feedforward architecture used is a significant simplification compared to the real gustatory system, which features abundant feedback loops both within taste buds and at the level of the brainstem and thalamus. These feedback connections play a crucial role in adaptation, habituation, and the modulation of taste perception by other modalities (e.g., olfaction).
3. **Influence of Other Sensory Modalities:** As noted in the introduction, taste is closely integrated with olfaction, somatosensory (texture, temperature), and even visual sensations. Our current model is purely gustatory and does not account for this multisensory integration, which is fundamental for forming the holistic perception of “flavor”.
4. **Learning and Plasticity:** Although we implemented an STDP-like rule for AMPA receptor phosphorylation, the learning mechanism was global and driven by an external genetic algorithm. In a real biological system, plasticity is local and distributed. Furthermore, the model lacks inhibitory interneurons, which are critical for forming contrastive and selective neural representations.

### 4.2. Robustness and Model Limitations

While our model relies on idealized biological parameters, we assessed the impact of potential biological variability on predictive accuracy through a sensitivity analysis [46]. We introduced random perturbations of ±20% to the synaptic weights and intrinsic neuron parameters to mimic the heterogeneity found in biological circuits [47]. Despite these perturbations, the network’s classification accuracy remained robust, decreasing by less than 10% on average. This suggests that the topological solution found by the genetic algorithm relies on coarse-grained features of the neural code (spike timing and population sparsity) rather than fine-tuned parameter values, thereby mitigating the limitations associated with parameter estimation in computational models.

### 4.3. Future Directions and Model Development

Overcoming these limitations opens several fruitful avenues for future research:

1. **Multisensory Integration:** The most evident development is the integration of an olfactory model. Following the same paradigm, a biomorphic model of the olfactory bulb could be created, where information is encoded by spatiotemporal spike patterns in glomeruli and mitral cells, and its output could be connected to the gustatory network. This would allow for the study of how combined taste-odor stimuli are encoded and discriminated at higher processing levels.
2. **Incorporating Feedback and Inhibition:** Extending the network architecture by including recurrent connections and populations of inhibitory interneurons (e.g., based on the Izhikevich model) would enable more complex and robust dynamic regimes, such as synchronization and competitive interaction (“winner-take-all”), bringing the model closer to its biological prototype.
3. **Deepening Intracellular Signaling:** The model can be made more detailed by incorporating comprehensive models of calcium dynamics and G-protein cascades for metabotropic receptors, using stochastic or deterministic systems of differential equations.

## 5. Conclusions

The development of computationally efficient yet biologically plausible models of taste perception represents a significant challenge at the intersection of computational neuroscience and neuromorphic engineering. This work addresses this challenge by proposing a novel hybrid modeling framework that bridges the gap between detailed biophysical representation and large-scale network simulation.

Our approach successfully integrates modality-specific receptor dynamics, Goldman–Hodgkin–Katz ion-current calculations, and detailed synaptic mechanisms featuring phosphorylation- dependent AMPA receptor trafficking and spike-timing-dependent plasticity. By combining the biological fidelity of Hodgkin–Huxley formalism with the computational efficiency of Izhikevich spiking neurons, we have created a scalable model capable of simulating gustatory transduction and neural coding processes.

The key results demonstrate that our optimized network can transform unstructured input representations into distinct, sparse, and reproducible spiking patterns that serve as neural fingerprints for different taste qualities. The genetic algorithm-based learning effectively tuned synaptic parameters to encode both taste pleasantness and discriminate between pure and mixed stimuli, showcasing the model’s capability for multimodal taste representation.

The importance of this research extends beyond theoretical neuroscience, offering practical implications for the development of neuromorphic gustatory systems. The model’s architecture provides a foundation for building energy-efficient electronic tongues capable of real-time taste analysis with biological fidelity. Furthermore, the framework establishes a basis for future integration with other sensory modalities, particularly olfaction, toward creating comprehensive flavor perception systems. It is also worth noting the potential for integrating such a model with memristors, similar to those used in olfactory models, through the use of synaptic plasticity [48].

While acknowledging current limitations in biophysical detail and network complexity, this work establishes a robust foundation for future developments in computational taste modeling. The proposed approach opens new possibilities for both understanding the neural basis of taste perception and engineering novel bio-inspired computing systems for chemical sensing applications.

## Data Availability

The raw data supporting the conclusions of this article will be made available by the authors on request.
